# UAV-Based and WebRTC-Based Open Universal Framework to Monitor Urban and Industrial Areas

**DOI:** 10.3390/s21124061

**Published:** 2021-06-12

**Authors:** Agnieszka Chodorek, Robert Ryszard Chodorek, Paweł Sitek

**Affiliations:** 1 Department of Applied Computer Science, Faculty of Electrical Engineering, Automatic Control and Computer Science, Kielce University of Technology, Al. 1000-lecia P.P. 7, 25-314 Kielce, Poland; a.chodorek@tu.kielce.pl (A.C.); sitek@tu.kielce.pl (P.S.); 2 Institute of Telecommunications, Faculty of Computer Science, Electronics and Telecommunications, The AGH University of Science and Technology, Al. Mickiewicza 30, 30-059 Krakow, Poland

**Keywords:** Internet of Things, artificial intelligence, unmanned aerial vehicle, WebRTC, real-time system

## Abstract

Nowadays, we are observing a rapid development of UAV-based monitoring systems, which are faced with more and more new tasks, such as high temporal resolution and high spatial resolution of measurements, or Artificial Intelligence on board. This paper presents the open universal framework intended for fast prototyping or building a short series of specialized flying monitoring systems able to work in urban and industrial areas. The proposed framework combines mobility of UAV with IoT measurements and full-stack WebRTC communications. WebRTC offers simultaneous transmission of both a real-time video stream and the flow of data coming from sensors, and ensures a kind of protection of data flow, which leads to preserving its near-real-time character and enables contextual communication. Addition of the AI accelerator hardware makes this system AI-ready, i.e., the IoT communication hub, which is the air component of our system, is able to perform tasks of AI-supported computing. The exemplary prototype of this system was evaluated in terms of the ability to work with fast-response sensors, the ability to work with high temporal and high spatial resolutions, video information in poor visibility conditions and AI-readiness. Results show that prototypes based on the proposed framework are able to meet the challenges of monitoring systems in smart cities and industrial areas.

## 1. Introduction

Nowadays, we are observing the rapid development of at least three emerging technologies, belonging to three different fields—first, Unmanned Aerial Vehicles (UAV) or drones, which recently left the military domain and have taken the civilian market by storm; second, the Internet of Things (IoT), which allows one to map real-world things in their digital representation; last but not least, the Web Real-Time Communications (WebRTC) [1,2,3], which introduces native, real-time media communication to the non-real-time normal Web environment.

### 1.1. UAVs and IoT

UAVs are used in many areas of life, both in the private and public sectors [4,5]. They collect and provide information or items (e.g., medicine or food) to hard-to-reach areas, especially in disaster areas. Drones also collect environmental data, e.g., to monitor pollution [6,7,8], to monitor parking lots [9] and road traffic [10,11,12], to monitor agriculture [13], and to assess the situation after a disaster [14,15]. The other area of applicability for drones is the delivery of parcels between two points [16]. The use of UAVs during the COVID-19 pandemic in China enabled the transportation of medical supplies to infected areas and the carriage of testing samples without using people [17]. During the time of the pandemic, there were discussions about using drones during and after a crisis caused by disease [18]. An unusual example of the use of drones during the pandemic was to display public health messages in the night sky [19]. Let us summarize this paragraph with The Commercial UAV News’ 2020 Report [20], which sees in drones a hundred billion market opportunities up to the end of 2020, forecasts "growing demand from the commercial and civil government sectors", and shows exterior inspections as one of the future-oriented areas of application.

Although anti-pandemic restrictions were a great opportunity for drone development [21], the UAV market merely tries to catch up with the second rapidly developing emerging technology—the IoT. The IoT market is now one of the most rapidly developing ones. It is estimated that around 20 billion IoT devices will be used in 2025 and around 500 billion in 2030 [22]. The secret of the success of the IoT lies in the design process of the IoT devices and systems, which are customized to the working environment and the collected data. Therefore, users of IoT devices or systems get complete solutions and need not worry about how to get the data from the sensor.

The fusion of UAVs and the IoT gives great results. The UAV is a platform enabling IoT devices to work in a given area [23,24], which is sometimes difficult to reach or too dangerous to allow people to enter. This is especially important when environmental conditions threaten human health (e.g., during radiation measurements in contaminated areas [14]). Some examples of communication networks comprised of UAVs and IoT devices are given in this paper [25].

### 1.2. WebRTC Technology and the Flying IoT System

A convenient user interface is the World Wide Web, so many Internet activities move towards the Web. One of these activities is the IoT. The Web of Things (WoT) is the integration of smart things with the Web [26]. However, classic Web, based on Hypertext Markup Language (HTML), is not able to provide native real-time communications. This was possible only when the HTML version 5 (HTML5) was introduced, which becomes a basis for the WebRTC technology [1,2,3].

The WebRTC is based on HTML5 and the JavaScript scripting language, and provides native real-time media transmissions in the non-real-time normal Web environment. WebRTC applications, although they are running in the run-time environments of Web browsers, show features of desktop-class applications, such as full interactivity, access to local input/output (I/O) devices (for example Web cameras, microphones, speakers, touchscreens), etc. The use of surveillance devices, especially cameras, allows the monitoring operator to observe given areas, which results in increased safety and security. Such a camera can be used, exemplary, for the surveillance of vehicles [27].

The WebRTC technology uses many sophisticated congestion control methods, such as sender-side TCP-friendly Rate Control (TFRC), the Google Congestion Control (GCC), and node-side stream replication simulcast and layered simulcast. Performance evaluation of the sender-side congestion control is presented in [28,29] and the node-side one in [30,31].

The WebRTC technology, although rapidly developed during the time of the pandemic, is not fully standardized yet. It is hoped that the standardization process of the first mature version of the WebRTC (so-called WebRTC 1.0) will be finished in the near future [32]. This raises problems related to the stability of the WebRTC specifications, especially with regard to security. Despite the inevitable teething pains, due to the advantages of WebRTC and the wide support by all leading browsers, this technology accounts for a considerable share of the market for Internet multimedia, and this market share has continued to increase sharply.

The convergence of the IoT and real-time communication is seen as an opportunity to build contextual communication [33], which puts real-time and associated non-real-time data in a common situational context. The paper [34] presents the convergence of the IoT and WebRTC in terms of the Quality of Service (QoS) of multimedia transmissions and the placement of middleboxes.

Flying IoT systems can use WebRTC technology on top of wireless communication, which allows them to meet the challenges of real-time communication, security and privacy, and contextual communication. The paper [35] considers the co-operation of the flying IoT system with three variants of a dedicated, custom Institute of Electrical and Electronics Engineers (IEEE) 802.11 (Wireless Fidelity, or WiFi) production network that differs in the infrastructure used (no intermediate access point, single intermediate access point, multiple intermediate access points). The paper [36] expands these considerations with the use of an existing, general-purpose network infrastructure.

The classic application of the WebRTC technology in IoT-based systems is to combine WebRTC-based real-time audio/video transmission with non-real-time IoT transmissions, done with the use of another technology. In particular, there are solutions that combine the WebRTC real-time communication and the classic Web of Things telemetry [37]. This solution uses only half of WebRTC’s protocol stack, i.e., protocols responsible for real-time media streaming, and the rest (responsible for data transmission) stays unused.

In our framework, we decided to use the full-stack WebRTC and, as a result, to transmit both media and telemetry using homogeneous technology. The main reason for choosing full-stack WebRTC was that it was designed and optimized for simultaneous non-reliable real-time transmission and reliable non-real-time transmission. This technology assures good co-existence of inelastic video traffic (which has stringent Quality of Service, or QoS, requirements), and elastic IoT traffic (which requires high reliability) in a shared link. This is important in terms of contextual communication, understood as in [33], which, in turn, entails the necessity of near-real-time transmission of IoT data. As was reported in the author’s previous paper [38], where the network conditions so permit, WebRTC gives sufficient QoS guarantees for both 4K video and IoT data transmissions, and faced with a conflict of interest between these two, protects the IoT data flow at the cost of the quality of the moving pictures. This effect would be difficult to achieve if heterogenous technologies were used.

The second reason for using WebRTC as the key technology for the proposed framework was the multi-streaming and multi-homing support offered by the SCTP. This enables relatively easy improvements of the system in the future, especially in terms of the reliability of IoT transmissions.

### 1.3. Motivations, Main Contributions and Organization of This Paper

The aim of this paper is to present an open, universal platform to monitor urban and industrial areas. The potential applicability areas of such a platform are the rapid prototyping of new solutions of flying IoT systems and the development environment for the rapid manufacturing of a short series of on-demand, custom flying IoTs. The latter are intended for use in emergency situations, such as infectious diseases, dangerous failures of industrial installations, and natural disasters.

The system proposed in this paper follows the general idea of the WebRTC-based flying IoT system, shown in [35]. The main contributions of this paper are:

- Building an open, universal, hardware-software flying platform, intended to be a basis for fast prototyping and/or manufacturing of a short series of IoT systems to monitor urban areas and providing exterior inspections of industrial installations. The structural building of this platform includes an air station and a ground one, connected via communication networks. The functional building includes a flying IoT carrier and a WebRTC-based IoT platform. The latter can be connected on-demand to a custom set of sensors and thus make a WebRTC-based IoT system.
- Building a communications hub for IoT devices mounted onboard the flying IoT carrier. This hub was built on the basis of a single-board computer and the author’s WebRTC software IoT broker, which implements Message Queue Telemetry Transport (MQ Telemetry Transport, MQTT) protocol.
- Building a twin communications hub at the ground station.
- Building an AI-ready air station (IoT communication hub), which is able to perform tasks of AI-supported computing.
- Evaluation of the whole monitoring system in terms of its ability to serve fast-response sensors, high temporal resolution of measurements, high spatial resolution of measurements, the ability to perform video monitoring in poor visibility conditions (insufficient sunlight, fog), and AI readiness.

The rest of this paper is as follows. The next, second section describes related work. The third section presents the architecture of the proposed framework, while the fourth section describes the system overview. The fifth section shows the system development, and the sixth section presents the prototype implementation. The seventh section discusses the results of laboratory tests and field trials carried out at the campus of AGH University, Poland. The eighth section discusses some use cases. The last, ninth section summarizes our experiences and concludes this paper.

## 2. Related Work

Traditional IoT devices are usually only sensors/actuators connected via the Internet to a cloud [39]. All processing is performed in the cloud [40] so that the IoT device can use CPU’s with limited computing power. Increasing usage of IoT devices and the necessity of processing (at least part of the data) in situ leads to moving the processing from the cloud to the edge of the IoT devices [39,40] to reduce traffic and latencies. Nowadays, this tendency also includes Artificial Intelligence (AI) and related fields, such as machine learning (ML).

Increased usage of ML in the processing of data coming from IoT systems initially only concerned cloud computing. The computing power of classic IoT devices is too small to provide ML computation in IoT nodes. To use machine learning without the cloud (required by time-sensitive applications), the use of edge computing is needed. Typically, edge computing is performed in the IoT hub.

There is a relatively small amount of literature that explores the combination of UAVs and WebRTC. Available sources often focus only on WebRTC-based transmissions of video streams from drone cameras [41]. UAVs were considered as air servers that enable WebRTC transmissions (airborne Web server and signalling servers for video and text chat transmission purposes [42]). WebRTC technology was used to assure UAV remote control [43].

The theme of the convergence of WebRTC with the IoT also remains under-explored in the literature. WebRTC was used for media streaming accompanying the application of specialized Web of Things technology [37]. The use of WebRTC for strictly IoT purposes was presented in papers [44,45], in which a non-real-time WebRTC DataChannel was extended with the ability for metadata transfer [44], or was a basis for building a rapid prototyping and development environment [45].

The various aspects of design and development of the UAV were presented in [46]. The UAV platform for experimental purposes is presented in [47]. Analysis of various flight controllers based on different types of MCU is presented in [48]. The UAV platforms used different types of MCU as the flight controller: Naza [47,49], Pixhawk [50], PIC [51].

Wide analysis of unmanned aerial systems (UAS) based environmental monitoring was presented in [52]. Aerial image acquisition using UAV for photogrammetric models of historical buildings and post-disaster documentation of historical places was presented in [53].

In recent years, ML, which uses deep learning (DL), has successfully been implemented in several domains [40]. Machine learning (ML) in IoT devices is used in data processing, management (e.g., resource allocation) [39] and communications [54].

Nowadays, we observe a tendency (Table 1) for the use of highly specialized drones to fall out of favor with regard to the use of low-cost consumer UAVs [55]. Moreover, quadcopter UAVs dominate the class of low-cost consumer UAVs. They can be factory-built as a series of drones, or can be built as customized devices, for the purpose of a specific usage, from ready-made components. The latter can also be built as “Do It Yourself” (DIY) drones.

In different areas of life, the use of low-cost consumer UAVs instead of factory-built ones is not trivial. Some of the issues to be tackled are: selection of the proper UAV, insurance of integration of IoT devices with the UAV, insurance of maintenance and service of IoT devices, power supply, communications, etc. [55]. Examples of frameworks that offer solutions suitable for the implementation of different types of UAV-based IoTs can be found in the literature.

Table 1 includes a comparison of, in the authors’ opinion, the most interesting frameworks that combine a UAV and the IoT. These frameworks are built on the basis of different types of aircraft, both fixed-wing ones and rotor-crafts (rotary-wing aircraft). From rotor-crafts, the most often used are hexacopters [55,56,58] and quadcopters [62,63]. Three frameworks are built as UAV swarms [56,58,59], and the rest were based on a single aircraft.

Frameworks listed in Table 1 were created for purposes of agriculture monitoring [4,57,60], air monitoring [55,59,63], monitoring of smart cities and the ocean [58], search and rescue missions [62], and for mobility assurance of a general-purpose IoT [4,23], including the collection of data from multiple IoT (due to co-operation with wireless sensor networks) [56].

The UAV framework for people and object detection using deep learning was presented in [62]. Raw video frames were processing using VPU. Detection of objects was provided using the OpenVINO library.

In the paper [55], a modular approach to the design of a UAV-based monitoring system was proposed. This system was based on a consumer UAV and was intended for monitoring air pollution. The described solution enables the use of different types of sensors. However, the system shown in the paper [55] has a number of limitations in terms of introducing new sensors. It uses a lot of programming solutions (C, Python, web application built on the Flask framework). In contrast, the proposed framework is based on one technology—the WebRTC—which makes it easier to implement.

The main advantage of the framework presented in the paper [4] is a lack of security issues. In this paper, multiple techniques and solutions aimed at assuring secure air-to-ground transmission from the UAV were presented. In our WebRTC-based framework, the required level of security is achieved by the comprehensive application of WebRTC technology - both video and IoT transmissions are cryptographically protected, and the aggregation of the video stream with the IoT data hinders a possible plaintext attack.

In the paper [61], a UAV fixed-wing platform was proposed for the collecting of data coming from environmental sensors. Measurements were made for the purpose of precision agriculture. In the case of this monitoring system, digital sensors sent data through a typical telemetry channel used in the UAV (two-way full-duplex 433 MHz serial UART telemetry link), and the solution typically used for the drone’s First Person View (FPV) transmissions (of analog Phase Alternating Line, or PAL, signal) was used for video transmission. Such a system is difficult to scale. In addition, it is difficult to transmit and to use data from the ground station to another destination.

## 3. Architecture of the UAV-Based and WebRTC-Based IoT System

The integration of an UAV and the IoT gives a flying IoT system, in which data gathered by IoT devices, mounted on a flying platform, are transmitted to the destination on the ground (e.g., to a cloud). In the case of such systems, decision-making processes are usually performed on the ground, away from the locations where events affecting these processes (e.g., emergency situations or natural disasters) take place. As an example, the flying IoT system delivers sophisticated and enriched information that goes to a higher level system like smart and sustainable cities and smart and sustainable infrastructure [64]. Flying IoT systems may be used at the level of the individual user, as well as at the level of society, government, or industry.

### 3.1. Structural Approach

**Figure 1:**
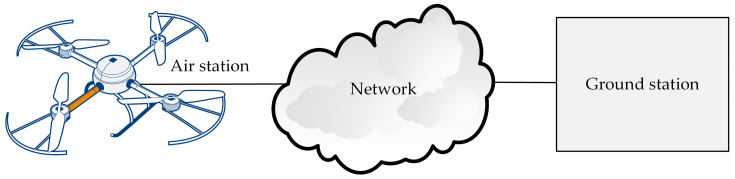
General concept of the structure of the UAV-Based and WebRTC-Based IoT system.

Structurally, the WebRTC-based flying monitoring system is composed of three elements: an air station, a ground station, and a network connecting the two (Figure 1). The air station gathers monitoring data (such as video from the drone camera, digital and analogue data from sensors, etc.), and sends them to the ground in real-time. If the computing power is sufficient to perform on-board processing, collected data can be processed in situ. Depending on the needs and necessary computing power, this may be from simple pre-processing to complex edge processing, including Artificial Intelligence.

**Figure 2:**
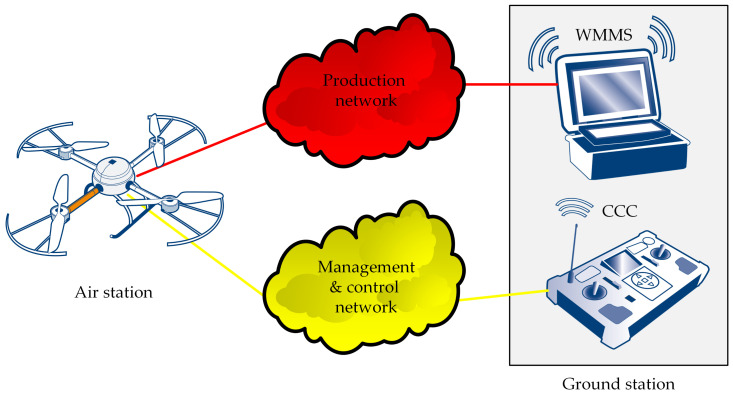
Structural diagram of the UAV-Based and WebRTC-Based IoT system: the two-devices and two-networks case.

The ground station performs two main functions: the online reception of the data transmitted by the air station and the sending of commands and control messages to the air station. This means that the ground station can be naturally implemented both as a single device (Figure 2) that performs both main functions, and as two separate ones (Figure 2): the WebRTC multimedia and monitoring station (WMMS), dedicated to the monitoring service, and the command and control console (CCC), used for piloting the UAV.

A single-device ground station is better in the case of the simplest monitoring systems, where the UAV operator also performs the task of the monitoring operator. Two separate devices should be used if the roles of the UAV operator and the monitoring operator are separated. In one-operator systems, if the UAV is equipped with an autopilot, a two-device ground station also can be used. The separation of the WMMS from the CCC can be used also for simplifying the system implementation.

The network connecting the air station and the ground station can be used for both transmissions of the remote control messages and transmission of the monitoring data (Figure 1). However, reliability issues mean that the carrier’s management and control network, used for piloting the IoT carrier, should be separated (Figure 2) from the production network, used for the transmission of monitoring data (both video monitoring and environmental monitoring). The use of these two networks avoids interference between two real-time high-priority sources of traffic.

### 3.2. Functional Approach

Functionally, the proposed system is composed of three co-operating subsystems: the flying IoT carrier system, the WebRTC-based IoT system, and the WebRTC management system.

#### 3.2.1. The IoT Carrier System

**Figure 3:**
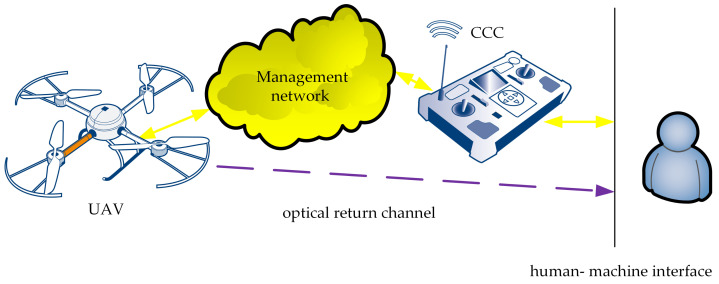
Block diagram of the IoT carrier system.

The IoT carrier system consists of three functional blocks (Figure 3): the IoT carrier, the management and control network, and the command and control console. In the case of our solution, the IoT carrier is a UAV able to carry the WebRTC-based IoT system to the desired destination. The CCC sends the UAV’s operator’s commands to the IoT carrier via the management and control network, and, in the opposite direction, data from the IoT carrier telemetry are sent.

A human IoT carrier’s operator communicates with the CCC using the human–machine interface built into the CCC device, which enables the operator to both pilot the flying IoT carrier and read the telemetry data. We assume that piloting the carrier requires flying within the line of sight. Gathering reasonably accurate visual information about the current situation needs the presence of custom human–machine interfaces, specific to the type of carrier being used. The optical return channel is just visual information, gathered through the observation of the flying carrier.

#### 3.2.2. The WebRTC-Based IoT System

**Figure 4:**
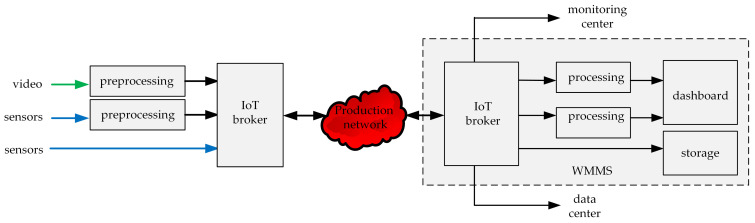
Block diagram of the WebRTC-based IoT system.

The main part of the WebRTC-based IoT system is the communication hub playing the role of the IoT broker, to which both sensors and a camera mounted on the IoT carrier can be connected. The general concept of the WebRTC-based IoT system is presented in Figure 4. Sensors connected to the communication hub may be IoT devices, and may also be raw sensors, digital, or analog. If sensors are IoT devices, they communicate directly with the IoT broker. Otherwise, they are connected to the communication hub which uses a part of its resources to build a virtual IoT device, which communicates with the IoT broker. Raw video data must be preprocessed before they are given to the broker input, and raw data coming from sensors are preprocessed if needed.

The WebRTC-based IoT system performs the monitoring service. The main functionalities of this system include (Figure 4):

- data gathering (video and environmental data), data preprocessing (if needed), multiplexing of environmental data in the flow, and the aggregation of the video stream and data flow in one aggregated stream—performed at the air station (the subsystem on the left side of the Figure 4),
- transmission of the aggregated stream through the production network,
- disaggregation of the received stream, demultiplexing of the disaggregated flow, processing (if needed), visualization, and (or) storing of received data for further processing—performed at the WMMS part of the ground station (on the right side of the Figure 4).

#### 3.2.3. The WebRTC Management System

The WebRTC technology requires access to three servers, used for WebRTC session establishment and maintenance purposes. They are: the traversal server, used for firewall and NAT traversal purposes, the Web server, from which the Web page containing the WebRTC application is loaded, and the signaling server, used for WebRTC session establishment and (if needed) renegotiation of parameters of the ongoing session.

**Figure 5:**
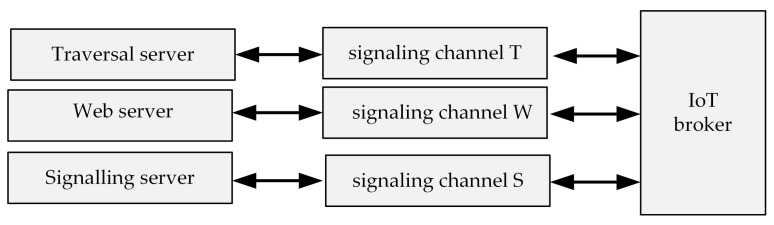
Block diagram of the WebRTC management system.

Each WebRTC-based IoT broker must be connected to these three servers (Figure 5). Any signaling channels, dedicated or shared, can be used to connect servers with an IoT broker. Servers may be publicly available or private, general-purpose or dedicated, located in the WebRTC application’s home networks or somewhere on the Internet. In the case of the proposed UAV-based and WebRTC-based open universal framework, we recommend using servers written as dedicated JavaScript applications, running on the WMMS with the use of the private node.js run-time environment. The signaling channels are then: a loopback interface (in the case of the IoT broker being a part of the WMMS) and the production network (in the case of the IoT broker running at the air station).

## 4. System Overview

**Figure 6:**
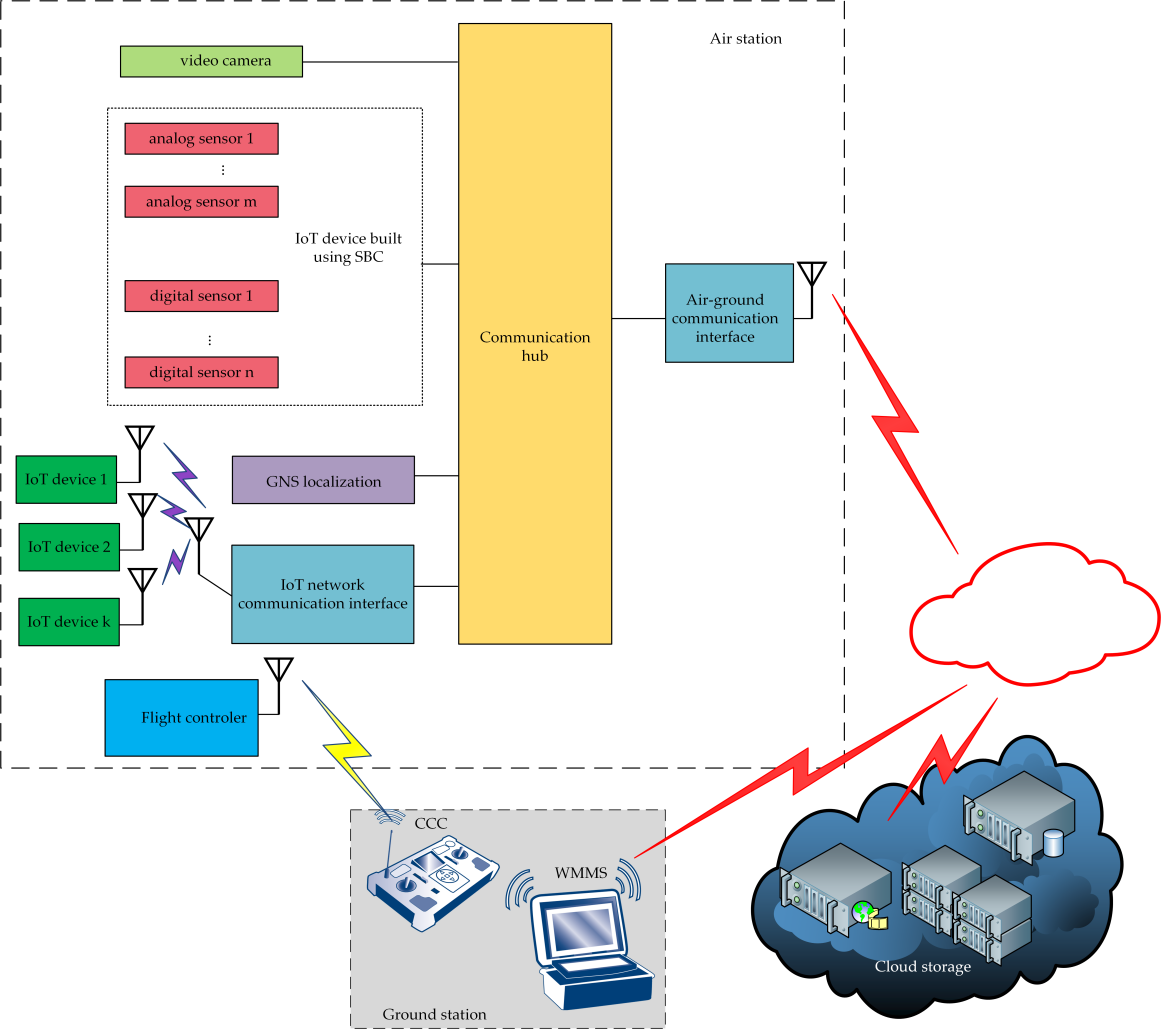
System overview.

The proposed UAV-based IoT framework is designed for fast prototyping or the building of a short series of IoT systems intended for the monitoring of urban areas and the inspection of industrial areas. These systems collect data coming from sensors measuring different quantities, generate a video signal that depicts a view of the external surroundings of the air station (which is an IoT and the UAV is the IoT carrier), and gather information about the current location, in which these quantities are measured and the video signal is generated. The gathered data from sensors and captured video information can be processed in situ, implementing the concept of edge computing, also using Artificial Intelligence. Gathered and, optionally, processed data are transmitted by the air station to ground systems, where received data can be visualized, processed and stored. A general view of the proposed framework is presented in Figure 6.

The central point of the air station is to be a communication hub, to which different devices are connected. The communication hub is built with the use of a single-board computer (SBC). IoT devices may be connected to the hub via the Bluetooth Low Energy (BLE) network or using any other network technology. Sensors that are not equipped with a central processing unit (CPU) can also be connected to the hub. Such sensors require the use of resources of the SBC (CPU, I/O) on which the software of the communication hub is run. Our software, which is a modification of our previous solutions [42,43], pre-processes the data coming from these sensors, and forwards them to the communication hub. Therefore, the virtual IoT device is created on the basis of the SBC resources and non-autonomous sensors mounted on board the air station. Such a virtual device is shown in Figure 6 as the IoT device built using the SBC block.

The communication hub integrates data coming from the sensors, a global navigation satellite system (GNSS), and video from the UAV camera in one, common session of air-to-ground data transmission. This functionality is assured by the WebRTC-based software—a WebRTC application of the IoT broker, which is run at the air station. This application uses the Message Queue Telemetry Transport (MQ Telemetry Transport, MQTT) protocol in the application layer of the Open Systems Interconnection (OSI) model and the Stream Control Transmission Protocol (SCTP) in the transport layer. The same application sends the video stream using the Real-time Transport Protocol (RTP). To assure time correlation between data coming from different devices, sensor data and GNSS data are multiplexed and sent in near-real-time. Near-real-time data flow and real-time video stream are aggregated in one stream (identified by one pair of IP addresses and one pair of port numbers).

Data coming from the air station are received by the WebRTC multimedia and monitoring station (WMMS), which is a part of the ground station. The received stream is disaggregated, and the data flow is demultiplexed. Received information is processed (if needed), visualized, and stored for further use.

The mobility of the WebRTC-based IoT is assured by the IoT carrier. In the proposed system, the carrier is a UAV that has airworthiness great enough to be able to perform measurements in different weather conditions, and its maximum load is sufficient to carry the IoT. This UAV is remotely controlled through the corresponding command and control console (CCC), which is the other part of ground station. For sake of safety and reliability, the UAV control transmission and the transmissions of video stream and sensor data are separated.

Data from IoT systems are often processed using machine learning algorithms. They typically are performed in the cloud, but, nowadays, it has been observed that there is a tendency to move that processing closer to the IoT devices. This is especially required for time-sensitive IoT applications. In that cases, the use of edge computing is needed.

Typically, edge computing is performed in the IoT hub. To improve the performance of our framework in ML tasks, the proposed framework includes AI accelerator hardware. There are several types of AI accelerators using dedicated GPU, VPU, or FPGAs [65]. For the proposed framework, an AI accelerator with good efficiency that cooperates with the TensorFlow library is recommended.

## 5. System Development

This section discusses the selection of the single-board computer, presents the integration of the SBC with input devices (sensors, UAV camera, auxiliary devices) and output devices (network adapters used for production network purposes), the use of the AI accelerator hardware, software development, the building of UAV-based IoT carrier, and system integration.

### 5.1. Single-Board Computer

A single board computer is an essential element of the monitoring system. The SBC runs WebRTC software and plays the role of the central element of the sensor network of the star topology, which is created with the use of the SBC, a UAV camera, and sensors intended for environmental monitoring. Therefore, the SBC must be able to assure both smooth pre-processing of gathered data, and real time or near real time re-sending of data to the ground station. The proper selection of the SBC, and good integration of the SBC with other elements and subsystems (such as input devices, production network, WebRTC software, and IoT carrier) is essential for proper functioning of the proposed framework.

For these reasons, the Raspberry Pi family of single-board computers was selected as the required class of SBCs. Because of its relatively high computing power and high energy efficiency, the Raspberry Pi family is the most-used hardware platform for IoT devices. Moreover, the Raspberry Pi SBCs are able to co-operate with both factory installed and external (connected through the Universal Serial Bus, or USB) network adapters, which makes this SBC more universal in terms of the range of supported production network standards.

Table 2 compares results of laboratory tests of the three currently most popular members of the Raspberry Pi family. Performance of Raspberry Pi single-board computers was measured with the use of the nmon tool [66]. All SBCs were loaded with three running software elements: Raspbian operating system, the Chromium browser working in headless mode, and the WebRTC application of the IoT broker. During tests, data were transferred from the SBC to the nearby WMMS. Results were averaged over three different sets of sensors, connected to the SBC. Average usage of memory and average CPU utilization was computed as an arithmetic mean, and maximum observed CPU utilization is the maximum value observed during all tests.

Performance metrics presented in Table 2 show that the Raspberry Pi 3 Model B (and it is to be expected that this applies to models older than this one) is not suitable for the purpose of the WebRTC-based universal framework because of its too small computational power, which results in high CPU loads. Observed CPU utilization was able to reach 100%, which resulted in the temporary freezing of the WebRTC application. The boundary model able to be used in the proposed framework is the Raspberry Pi 3 Model B+, which was successfully used as a communication hub during experiments presented in the papers [35,36]. However, this SBC works close to the limits of its capabilities (although it does not exceed them—at least during tests shown in Table 2), while running the WebRTC application of the IoT broker.

The last of the tested models, the Raspberry Pi 4 Model B, has enough computational power to be used in the tested hardware configurations, and its computing power reserve is large enough to also serve more complex hardware configurations. This is the result of a slightly faster Central Processing Unit (CPU) and an improved Graphics Processing Unit (GPU), as compared with the older model.

Other improvements introduced to this SBC, which can be useful in the case of the proposed framework, are more efficient USB ports, and new connectors (e.g., a High Definition Multimedia Interface, or HDMI). The ability to use more memory (up to 8 GB of factory installed memory) and the new USB Type-C port (instead of micro USB type B), which increased the maximum power that can be delivered to the SBC from 12.5 W to 15.3 W, may also be important in future use.

As a result, the Raspberry Pi 4 Model B was selected as the hardware platform of the communication hub.

### 5.2. Integration of the SBC with Input Devices and Output Devices

**Figure 7:**
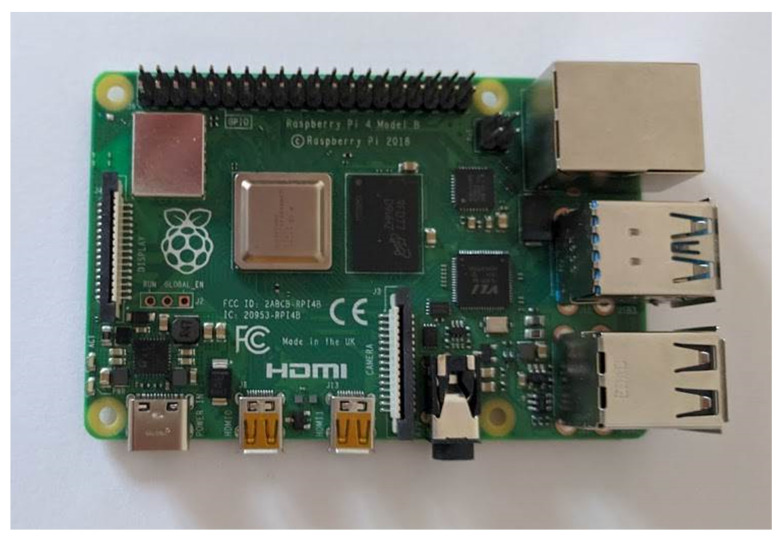
The Raspberry Pi 4 Model B single-board computer used in the proposed framework.

The main element of the Raspberry Pi 4 Model B (Figure 7) is the Broadcom BCM2711 quad core processor, working at the clock frequency of 1.5 GHz. The Broadcom BCM2711 implements ARM Cortex-A72 microarchitecture, which, in turn, implements the ARMv8-A 64-bit instruction set. Generally, this processor is able to co-operate with 1 GB, 2 GB, 4 GB, or 8 GB of LPDDR4-3200 synchronous dynamic RAM (SDRAM). In the case of the proposed framework, the 2 GB SDRAM was installed.

The Raspberry Pi 4 is equipped with several built-in interfaces for intra-system and inter-system communications. Most of them found their application in the integration of the SBC with input devices and output devices. They are:

- 2.4 GHz and 5.0 GHz IEEE 802.11AC network interfaces (for communication with the ground station),
- Bluetooth 5.0 BLE (Bluetooth Low Energy) network interfaces (for wireless connections with sensors),
- two USB 3.0 ports and two USB 2.0 ports (for connecting UAV cameras),
- one 40-pin General Purpose Input–Output (GPIO) connector (for wired connections with sensors).

Each of the pins of the GPIO is configured to one of four modes of operation: digital, Inter-Integrated Circuit (I${}^{2}$C), Serial Peripheral Interface (SPI), or Universal Asynchronous Receiver/Transmitter (UART). The mode of the GPIO pins depended on the output interfaces of the connected sensors, and the pins are set to the proper mode by the WebRTC application.

**Figure 8:**
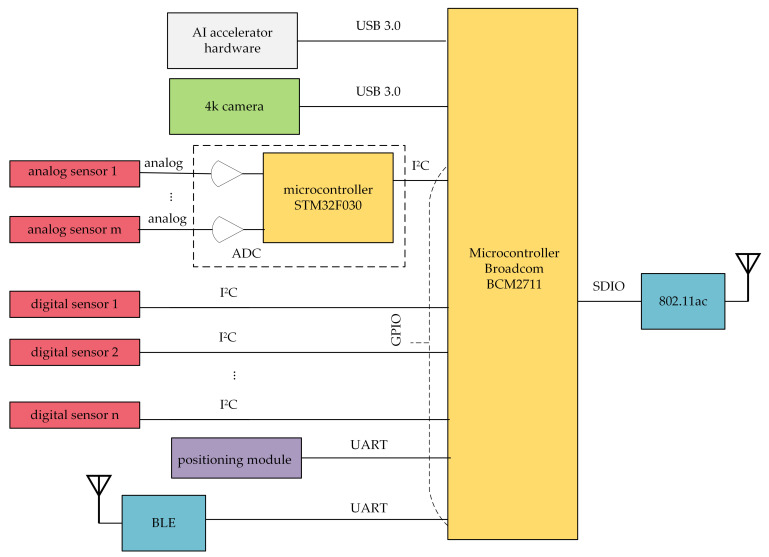
Integration of the SBC with I/O Devices.

Figure 8 presents a schematic diagram of the SBC with connected input (left side) and output (right side) devices. The Broadcom BCM2711 microcontroller with WebRTC software running on it acts as a communication hub for both the UAV camera, positioning module, and sensors. The 4K camera, mounted on a gimbal (controlled via the CCC), was connected to the SBC through one of the USB 3.0 ports. Digital sensors are directly connected to the Broadcom BCM2711 processor using the I${}^{2}$C serial bus. Measurement signals coming from analog sensors go to the Broadcom BCM2711 through an analog to digital converter (ADC), which is based on the STM32F030 32-bit Microcontroller Unit (MCU). The MCU is a low-power ARM Cortex M0 with 16 kB Flash memory, 16 kB Static RAM (SRAM), two I${}^{2}$C interfaces, 11 timers, 55 fast Input/Output (I/O) ports, and 8-channel 12-bit ADC. The internal ADC has conversion ranges from 0 V to 3.6 V. The STM32F030 microcontroller is connected to the Broadcom BCM2711 using the I${}^{2}$C serial bus.

To store the spatio-temporal context of the transmitted data, measurements coming from the sensors must be associated with time and location metadata. While time can be easily received from a system clock, the gathering of the current position of the flying IoT needs the presence of a positioning system. Because there is no connection between a UAV’s flight controller and the Broadcom BCM2711 microcontroller, the position from the on-board equipment cannot be transferred to the IoT. Therefore, the set of sensors must be accompanied by a positioning module that will be connected to the SBC in a device-specified manner. In Figure 8, the exemplary positioning module is connected to the SBC via a GPIO pin set to the UART mode.

Multimedia data from input devices are processed by the WebRTC application, multiplexed and aggregated, and then sent to the ground station. The Raspberry Pi 4 single-board computer is factory equipped with an IEEE 802.11ac dual band (2.4 GHz and 5 GHz) network interface, connected to the Broadcom BCM2711 microcontroller through the Secure Digital Input Output (SDIO) interface. It was used to assure connectivity between the air communication hub and the WMMS.

### 5.3. AI Accelerator Hardware

IoT hubs that perform edge computing are more and more common nowadays. To increase the efficiency of edge computing, the proposed framework was equipped with AI accelerator hardware. AI accelerator hardware is produced in a form of a USB stick and is recommended to be connected to the SBC through a high-speed interface USB 3.0. This allows the proposed communication hub to be assured of adequate performance for both WebRTC and IoT.

Generally, operations that use AI are carried out in several phases, two of which (machine learning and the execution phase) can be supported by the AI hardware accelerator. In all of the applications that require large computing power, due to large power consumption and (or) the long duration of the operations, the recommended method for the use of the AI hardware accelerator is to perform the machine learning phase (especially ML for supervised deep learning) in an efficient stationary computing system, e.g., in a specialized cloud environment, whereas the implementation of the second phase (execution phase) is carried out locally, with the use of the proposed framework.

In the case of the proposed framework, the model created remotely in the machine learning phase is sent to the framework and run locally. Thanks to this, an external system (e.g., a specialized cloud) carries out this phase of the machine learning, and the resulting model can be sent to a very large number of devices and can be executed directly by them. This allows the system to be fully scalable. The model can be modified periodically on the basis of the data sent by the air station in successive measurements or, if multiple air stations are used for monitoring of the same item, on the basis of data sent by individual air stations.

**Figure 9:**
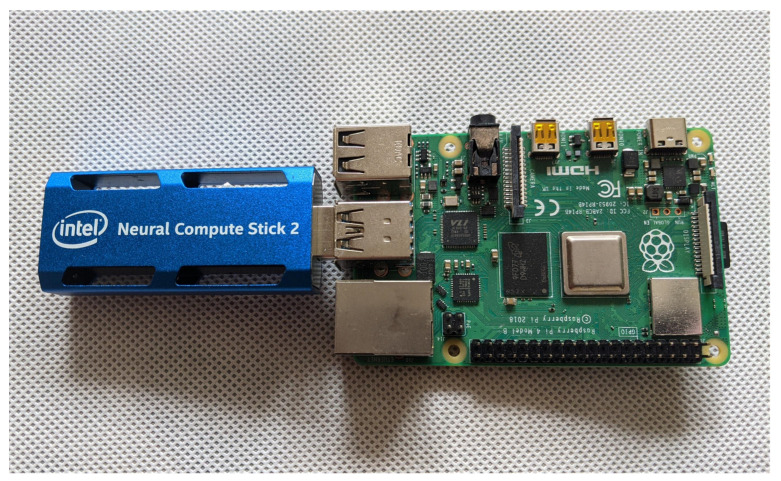
The Raspberry Pi 4 Model B single-board computer with attached AI accelerator hardware.

We have built into the framework mechanisms for importing and exporting the model used by AI. As an example of an accelerator hardware, the Intel Neural Compute Stick 2 (NCS2) was used (Figure 9). This hardware accelerator is characterized by high efficiency with the lowest power consumption of the available popular systems. The power consumption, measured during work in the proposed framework, was from 0.96 W to 1.51 W.

### 5.4. WebRTC-Based Software

The WebRTC-based software is a revised and simplified version of the WebRTC-based application of the IoT broker, written in the JavaScript scripting language, which was developed based on the need of the research reported in the paper [34] and then adapted to a flying monitoring system presented in the paper [35].

The developed software is used for both data gathering and for communication between a station that collects the data and a station that visualizes and stores received data. The base software is a Web application written according to the concept of the Single Page Application (SPA). This application combines the functionality of an IoT hub and an IoT broker. It enables the ability to control the measurement process in the air station (for sensors connected directly to the SBC, here: to the Raspberry Pi microcontroller). The application running on the ground station enables real-time data presentation (on a dashboard) and data storage locally or in the cloud.

Compared to the previous solutions, the current solution is fully flexible. It enables the easy modification of the software according to the hardware configuration being used. AI support has been added. Some functionalities (e.g., integration with the flight controller) were replaced by more simple solutions that require less computing power (such as the use of a dedicated GNSS device). The information flow in the air station has been modified.

The application is transmitting both video from a UAV camera and IoT data (both data from sensors and spatio-temporal metadata) between the air station, where the video information is captured and IoT data gathered, to the ground one, where transmitted information is visualized on the dashboard and stored for further processing and analysis. Video is transmitted as WebRTC’s media stream, using the RTP protocol via the RTCPeerConnection application programming interface (API). IoT data are transmitted inside messages of the MQTT protocol, as WebRTC’s non-media flow, using the SCTP protocol via RTCDataChannel API.

The WebRTC application used in the proposed framework is built using a single-page application (SPA) web programming paradigm. To enable work on both the air station and the ground one, the application is run in one of two modes that are set as Uniform Resource Locator’s (URL’s) query string parameters. These modes are:

- the UAV mode, intended for the application that is run on the SBC (at the air station),
- the WMMS mode, as the name suggests, intended for the application that is run on the WMMS part of the ground station.

**Figure 10:**
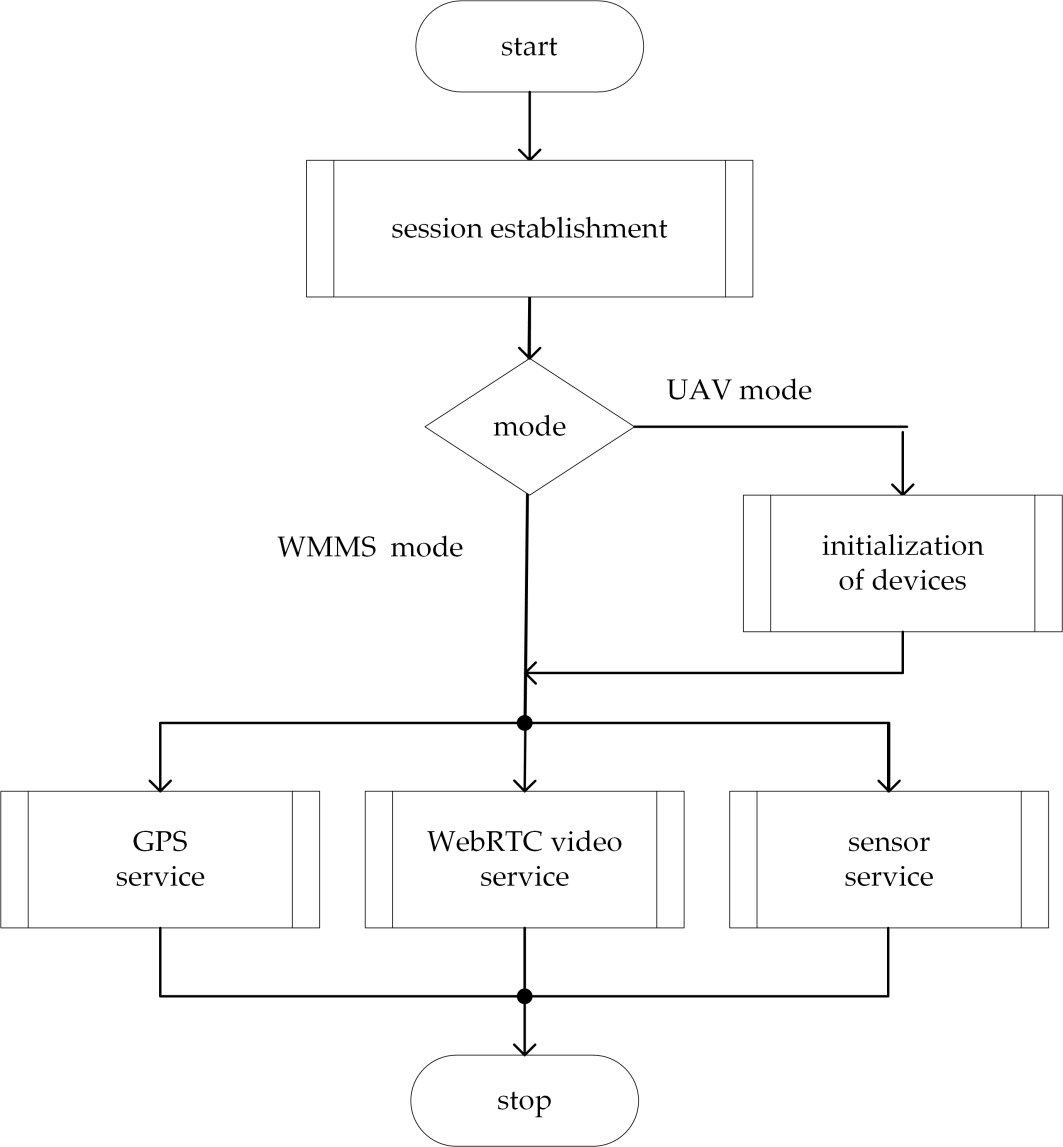
Overview of the WebRTC application.

The general principle of the operation of the WebRTC application running in UAV mode is illustrated in Figure 10. The program begins with the WebRTC session establishment and initialization of devices. During session establishment, applications running in UAV mode and in WMMS mode exchange session information, register user-defined procedures of the video service, and finally establish direct, peer-to-peer communication with each other. Initialization of devices is made only by the WebRTC application running in UAV mode. It includes initialization of local GNNS and sensors and setting the GPIO pins to modes required by particular sensors. These operations are device-specific and must be done according to devices’ manuals. To facilitate initialization, the WebRTC API was supplemented with the zkgpio library.

When a session is established and devices are initialized, three services are performed in parallel: the GPS service, the WebRTC video service, and the sensor service. In the case of the WebRTC application running in UAV mode, these services are focused on data collection. The GPS service consists of the cyclic reading of the positioning module, and then the current position of the IoT carrier is sent to the ground station. The sensor service cyclically polls the sensors connected to the SBC, reading current measurement if the sensor is ready, and sends the current measurement datum associated with current time datum coming from the system clock. In these operations, the WebRTC API was aided by the zkgpio library. The video service includes compression, encoding, and real-time streaming to the ground station of video information captured from the UAV camera. This is entirely done using the WebRTC API.

**Figure 11:**
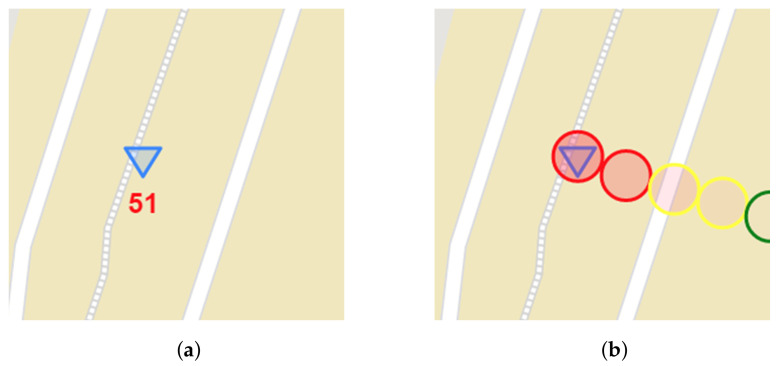
Marker of current position of the air station and additional information drawn on the map: (**a**) text label (current concentration of exhaust gases in ppm); (**b**) markers of air quality.

The WebRTC application running in WMMS mode starts with session establishment followed by three services performed in parallel that receive information from the air station, visualize it on a dashboard, and store it for further use. The GPS service shows the current UAV position as an inverted triangle drawn on the map of the traveled area displayed on the dashboard in a fixed size window (Figure 11). The map is downloaded from the OpenStreetMap (OSM) repositories and processed with the use of the Leaflet library. The other fixed size window allows the WebRTC video service for the presentation of scaled down video. The full-size video may be, optionally, displayed on an external monitor connected to the WMMS computer device. The sensor service plots current measurement data at the last position of a proper time graph. Due to the limited area of the dashboard, only up to three graphs, selected by the WMMS operator, are shown and only measurements collected during the last 40 s can be presented on each time graph. To visualize and (or) analyze older data, spatio-temporal metadata and video information, one should refer to the data stored on the disk or in a cloud.

To easily identify problematic areas, information about current air quality can be drawn on a map, on the flight route. This information may be presented as a text label (Figure 11a), which moves with the marker of the current position of the air station. It may be also shown in the form of circular markers, where colors of circles show good (green), medium (yellow) or bad (red) air quality (Figure 11b). In order not to obscure the picture, the circles disappear from the map after a while.

### 5.5. The IoT Carrier and System Integration

This section includes a selection of the IoT carrier, including a selection of an aircraft and a flight controller, and some practical aspects of system integration, such as selection of the CCC, power supply, attachment of the IoT system to the IoT carrier, operating systems, and run-time environments proposed to be used in this framework, and the WMMS.

#### 5.5.1. The IoT Carrier

Although there are no special assumptions imposed on UAVs serving as IoT carriers, the assumed openness of the proposed framework entails that the UAV that will assure mobility of the WebRTC-based IoT also should be, as far as possible, of an open and universal construction. Because the proposed framework is intended for monitoring urban and industrial areas, such features as low speed, ability for following complex trajectories, and maintaining the required flight altitude with high accuracy (including hovering at the designated position) seems to be essential. Last but not least, the UAV should be characterized by sufficient airworthiness to conduct measurements in different weather conditions. As a result, the choice of the UAV was guided by three assumptions:

- the UAV should be an open hardware platform,
- the UAV should be characterized by both good adaptability for carried equipment and suitable airworthiness,
- to assure suitable mobility, the UAV should be a multi-rotor rotorcraft.

A good trade-off between the cost of a rotorcraft and its operational properties has decided that, from several often used types of multi-rotor rotorcrafts (from tricopters to octocopters), a quadcopter was selected for use in the proposed framework. Quadcopters are simple, popularly used, of relatively cheap constructions, characterized by relatively high reliability and maneuverability.

**Figure 12:**
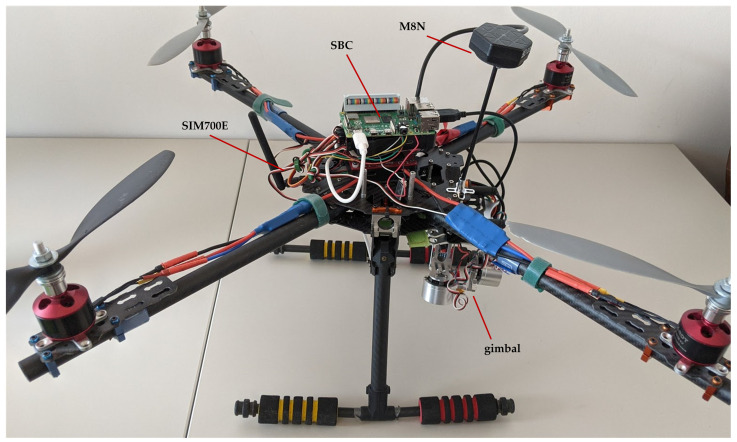
The air station is ready-to-fly. The green board on the top is the SBC, the hexagonal device is the M8N GPS SE100 positioning module, and the thick antenna on the left belongs to the SIM7000E positioning module. The shiny cylinder under the right arm (in the front) is the gimbal.

The quadcopter chosen to be the IoT carrier was customized to fit tasks specific for weather and pollution measurements in urban and industrial environments. To assure openness of the structure and achieve good airworthiness, it was built on the Tarot FY650 isosceles frame (650 mm tubular arms—Figure 12), which was driven by four Tarot 2814 700Kv2 motors equipped with HobbyKing 30A/40A BEC ESC speed controllers. The unladen weight of the ready to fly UAV is about 1.7 kg, and the maximum load is up to 1 kg. The take-off weight of the air station with the technology demonstrator mounted on board was about 1.95 kg.

#### 5.5.2. The Flight Controller

The Pixhawk general purpose flight controller was selected to control the UAV, due to its openness and universality. This open-hardware flight controller, which supports open source control software, in the proposed framework is used for both remote control of the UAV’s movements and remote control of the movements of a gimbal, on which a 4K camera is mounted. As the control software, the ArduPilot open source autopilot [67,68] was used. The flight controller allows the flight route to be defined ad hoc (manually, using the CCC) or as pre-defined trajectory (using a planner tool). The ArduPilot supports the Mission Planner software tool, used for the planning of autonomous missions. For mission planning, open maps downloaded from the OpenStreetMap repository are used.

The Pixhawk controller used in the proposed framework had several factory installed sensors, such as an accelerometer, a barometer (used as a barometric altimeter), a gyroscope and a magnetometer. It co-operates with external positioning and communication subsystems. In the proposed framework, the M8N GPS SE100 positioning module was used. This device is a functional equivalent of the SIM7000E positioning module, used by the IoT system. The communication subsystem, co-operating with the Pixhawk, must be selected together with the command and control console.

#### 5.5.3. The Command and Control Console

For remote control of the UAV, the FlySky FS-i6X command and control console were chosen. For connectivity between the Pixhawk and the CCC, the FlySky FS-iA10B receiver of the RC system was selected. The couple FlySky FS-i6X and FlySky FS-iA10B enables bidirectional communication in 2.4 GHz band with the use of 10 channels. The number of channels is large enough to assure transmission of both telemetry and control data (both UAV control and gimbal control). The use of a Frequency-Hopping Spread Spectrum (FHSS) 2.4 GHz radio with AFHDS 2A protocol reduces interferences from other transmitters.

The FlySky FS-i6X command and control console has open-source firmware, which may be modified and flashed.

#### 5.5.4. Power Supply

The described IoT carrier should be powered by a Lithium Polymer (LiPo) battery with a three-cell (3S) to five-cell (5S) pack, where 1S is 3.7 V, which gives nominal voltages from 11.1 V to 18.5 V. Smaller voltages, offered by LiPo 1S and 2S, are not useful for such large UAVs. The described prototype was equipped with a four-cell (4S) battery pack, which lasts more than 20 min of flight during bad weather (cold, strong wind) and up to 30 min in good weather conditions (warm, calm). To provide the required power (with a voltage of 5 V) to the Pixhawk flight controller, a battery eliminator circuit (BEC) is used. The BEC converts higher voltage from LiPo to the required 5 V and eliminates interferences from high current brushless motors. This voltage is also used to supply the Raspberry Pi single-board computer.

#### 5.5.5. Attachment of the Single-Board Computer and Sensors

The single-board computer and sensors are attached to the UAV. To avoid vibration stress, the SBC and almost all sensor devices (except the gas sensor) are mechanically attached to an anti-vibration platform with bolts, nuts and plastic spring washers. The anti-vibration platform is a mounting plate attached to the UAV deck through vibration dampers. As the vibration dampers, usually rubber or silicone ball dampers or anti-vibration foam tape are used. In the case of the proposed framework, rubber dampers were applied. To assure accurate results of air quality monitoring, the gas sensor was hung on electrical wires ending in a plug that was inserted into a socket with a latch. Because there were no additional strings, wires connecting the gas sensor with the SBC both transmit information about gas concentration and mechanically attach the sensor to the ADC board.

As was mentioned above, the power supply of the single-board computer (and via SBC also powering the sensors) is assured by the IoT carrier. The SBC has its own, separate from the flight controller’s BEC, power converter 2–6 s lipo battery to 5 V. It improves reliability and reduces power supply noise.

#### 5.5.6. SBC: Operating System and WebRTC’s Run-Time Environment

The single-board computer works under the control of the Raspbian operating system, which is a modification of the well-known Debian distribution of the Linux operating system, intended for the Raspberry Pi. There are several operating systems able to run on the Raspberry Pi—just to mention the more popular ones: Open Source Media Center (OSMC), RISC OS, Raspbian, RaspBSD, Ubuntu Core, Windows IoT Core. However, Raspbian is probably the most popular one, and the kernel version, which was used in the presented prototype (initially, 4.19.42, then 5.4 LTS), is long-term (i.e., a version with long-term support - the Linux kernel 5.4 LTS will be supported until the end of 2025) version, which is considered very stable. To save memory and energy, the Raspbian does not use the graphical user interfaces (GUI).

Over the Raspbian operating system, the Chromium browser was run without calling GUI functions (in headless mode). The Chromium is the Linux version of the Google Chrome browser. This browser was used as a run-time environment for the WebRTC application working in UAV mode.

Both the Raspbian and the Chromium are open-source software.

#### 5.5.7. The WebRTC-Based Multimedia Monitoring Station

As the WMMS hardware, any stationary, portable or mobile computer device—from desktop computers, through laptops, to smartphones—and any popular operating system can be used. The choice of a computer device should be guided by the comfort of use and the performed tasks. When choosing an operating system, one should be guided by the existence of a WebRTC-capable browser able to be run on this system.

During experiments, a Dell E6430 laptop was used. This device is equipped with MIL-STD 810G tested casing, which allows operators to perform monitoring in different weather conditions, and a 14″ anti-glare LED display, which enables work in any lighting conditions, including full sunlight. A quad core (eight threads) CPU and 16 GB of RAM were able to assure good work of the WMMS. The laptop worked under the control of the Windows 10 Pro operating system, on which the Chrome browser was run. Google Chrome is considered one of the reference browsers for WebRTC. This browser was a run-time environment for the WebRTC application working in WMMS mode.

## 6. Exemplary Prototype Implementation

As an example of the prototype based on the proposed framework, a weather monitoring system was used. The reasons for the selection of weather monitoring as an exemplary system were a plethora of low-cost sensors for the measurement of environmental parameters and the importance and commonness of measurements of environmental parameters. They are used both as main data and metadata associated with the measured value of the main quantity, as raw data and processed data (e.g., using Artificial Intelligence (AI) driven weather forecasting systems). Weather measurements are used for calibration of other sensors for environmental monitoring, such as air quality sensors. Last but not least, there are some implementations of efficient AI-driven weather forecasting algorithms, able to be run on the Raspberry Pi, which can be used for evaluation of AI-driven on-board processing.

The prototype air station of the weather monitoring system includes ten sensors of different types that are able to measure a couple of environmental parameters. Detailed types of sensors used in the experiments can be found in Table 3. Each of the selected sensors is able to measure temperature. Almost all of these sensors (except the BMP280) also measure humidity. Measurements carried out by the BMP280 and the BME280 include atmospheric pressure.

**Figure 13:**
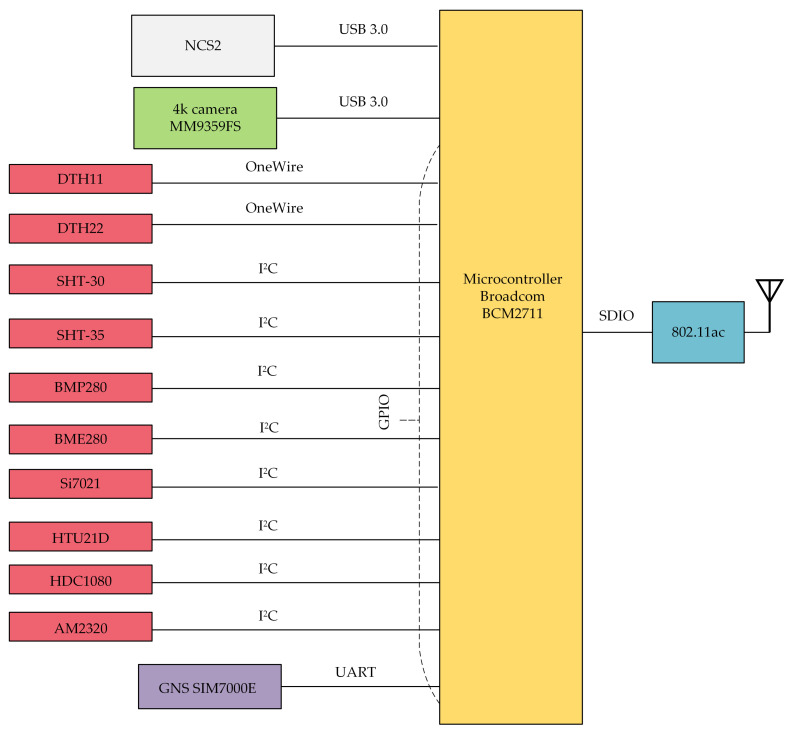
The exemplary prototype.

All selected sensors are directly connected to the SBC (Figure 13), overwhelmingly through the I2C interface. Only the DTH11 and the DTH22 sensors were connected through the OneWire serial interface. Wired connections assure both bidirectional communication between the SBC and a sensor, and the power supply of a sensor.

The set of sensors was supplemented with the Waveshare SIM7000E NB-IoT LTE GPS HAT positioning module. This device includes three navigation satellite systems (NSS), and a fusion of information coming from independent NSSs allows the module to calculate location with an accuracy of up to 0.5 m. The NSSs used by this positioning module are: the GPS (Global Positioning System), the GLONASS (Globalnaya navigatsionnaya sputnikovaya sistema—in Russian; literally: global NSS), and the BeiDou (in Chinese: the Great Bear, or Ursa Major, constellation). To connect the Waveshare SIM7000E module to the microcontroller, a GPIO pin set to the UART mode was used.

It is worth noting on this occasion that the prototype was equipped with the Waveshare SIM7000E positioning module, which is also a Long Term Evolution (LTE) network adapter. Although the LTE assures coverage of relatively large areas, in our experiments, the Waveshare SIM7000E was not used for data transmission and worked only as a positioning module. This is because preliminary tests of our prototype showed that the LTE is less suitable for air-to-ground 4K video transmissions than the IEEE 802.11ac. These findings were confirmed by the conclusions resulting from the paper [69], which analyzes air-to-ground transmissions from a UAV. Simulation results presented in [69] showed that Full HD 1080p 30 fps video can be transmitted via an LTE network with suitable Quality of Experience (QoE) only in a good condition channel. This suggests large difficulties (reflected in our experiments) in transmission of 4K video via an LTE network with sufficient quality. To achieve better coverage of an IEEE 802.11ac network and greater range in urban areas, during experiments, the 2.4 GHz frequency band was used.

Besides devices connected to the SBC through the GPIO, such as weather sensors and the positioning module, devices connected via USB were also used, such as the 4K camera and the AI accelerator hardware (Figure 13). As the 4K camera, the Manta MM9359FS was chosen, due to its resistance to vibrations and environmental conditions, and, last but not least, the possibility of streaming the signal via USB.

To build a complete IoT hub with edge computing for ML, the AI accelerator hardware was added to the exemplary prototype air station. As an accelerator hardware, the Intel Neural Compute Stick 2 was used, selected for this purpose due to its ability to co-operate with the TensorFlow library. This library was used in one of the experiments described in the next section.

## 7. Results and Discussion

The presented open universal framework for rapid prototyping of UAV-based monitoring systems was empirically evaluated in terms of readiness for small response times, high temporal and spatial resolution of measurements, video information in the context of measured data, and AI readiness. As an example of this system, a weather monitoring system, assembled using the proposed framework and several low-cost sensors, was used. Experiments were carried out at the campus of the AGH University of Science and Technology, Poland. For the sake of comparison, environmental measurements done by the air station were accompanied by environmental measurements done by fixed sensors of the SBS-WS-400 weather station, which are permanently attached to the roof of a building of the Institute of Telecommunications of the AGH University, Poland.

### 7.1. Response Time

The first experiment consisted of a series of measurements carried out in a laboratory of the Department of Telecommunications of the AGH University, Poland. During the normal work of the prototype, the set of ten sensors was cyclicly pooled by the communication hub. Sensors used in the tested prototype were selected in terms of different nominal response times (given, e.g., by the manufacturers in the sensor specifications).

The experiment was carried out in series of 100 measurements, which finished when all sensors answered 100 times. After each series, the air conditioning was set to a new value. A new series of measurements began when the condition of the air in the laboratory achieved set-point values. During experiments, both the WebRTC video service and the GPS service was switched on, so the SBC was loaded as during normal work. Results of the first experiment are summarized in Table 4 and Table 5.

Although sensors were requested in one hub’s message for both temperature and humidity, response times observed in the case of each measured quantity were usually different, which is caused by the independent update of the two registers intended for instantaneous storing of these two values. These differences also are visible in nominal response times. To enable easy comparison between the nominal response time and the corresponding statistical items, the rightmost columns of Table 4 and Table 5 include the value of response time taken from manufacturers’ specifications.

As is shown in Table 4 and Table 5, the WebRTC application running on the Raspberry Pi 4 Model B, selected as the hardware platform for the communication hub, was able to serve each of the tested sensors, even the Si7021 sensor, with the fastest sampling rate of all sensors used in the prototype.

However, nearly half of the sensors (4 of 10) listed in Table 4 and more than half of the sensors (5 of 9) listed in Table 4 never achieved a sampling rate declared by their manufacturers. This includes all tested slow-response sensors (with response times of seconds), which in the best case responded 5% or 10% slower than was reported in their specifications, and the SHT series of fast-response sensors (with response times of milliseconds), which responded at least 2% slower (SHT-30, only when humidity measurements were performed) or 7% slower (SHT-35, during both temperature and humidity measurements) than their nominal response times.

As is presented in Table 4 and Table 5, the maximum measured response times always exceed the corresponding nominal response times, which indicates that not one of the tested sensors was able to perform long-term stable work at its nominal sampling rate. An analysis of the row data shows that response times have a tendency to cluster near both extremes. This means that the arithmetic mean is situated more or less in the middle of the range defined by the minimum and maximum. This also means that, even in the case of large differences between the maximum response time and the nominal one, which can reach 75% (the Si7021 sensor carried out temperature measurements), maximum values of response time meet the three-sigma criterion.

Generally, the smallest relative differences between the maximum response time and the nominal one were observed in the case of slow-response sensors (15% or 20% in the case of temperature, 20% in the case of humidity). In the case of fast-response sensors, this difference is greater and amounts to about 25–45% of the nominal response time. However, single cases of fast-response sensors where the maximum response time are outside this range also can be found (e.g., the HDC1080 sensor).

In most cases, the mean response time was about 10% to 20% greater than the response time included in the sensors’ specifications. The smallest relative difference between the mean response time and the nominal one (4%) was observed in the case of the BME280 sensors. The largest relative differences were observed when the Si7021 sensor carried out temperature measurements (46%), and when the HTU21D sensor performed humidity measurements (25%). It is, however, important to keep in mind that these percentage differences are related to the shortest nominal response time listed in Table 4 (Si7021: 2.4 ms), and to the third shortest nominal response time listed in Table 5 (HTU21D: 4 ms).

It is also worth remarking that the results of the measurements do not show a correlation between the measured response time and the temperature in the laboratory, at least in the applied range of air conditioning set-points.

### 7.2. High Temporal Resolution

The results obtained during the first experiment show a response time for requests coming via the I2C interface. Reaction to rapid changes in environmental parameters is the subject of the second experiment. This experiment depends on the flying of the air station through areas of expected weather conditions (different or the same during a whole flight). As the air station, the prototype was used.

After take-off, the air station was positioned over the start point, waiting for the stabilization of the results of measurements, and then began to move in a straight line. The air station was traveling at an altitude of five meters (the lowest one recommended for use in areas where the presence of people should be expected). The length of the flight route was 200 m. The speed of the air station (0.7 m/s) and the duration of a single pooling cycle (2.5 s) were adapted to the sampling rate of the slowest sensor (the DHT22, with the maximum response time of 2.4 s). During each flight, parallel measurements of environmental parameters were made. The low-cost sensors used for experiments had factory calibration.

**Figure 14:**
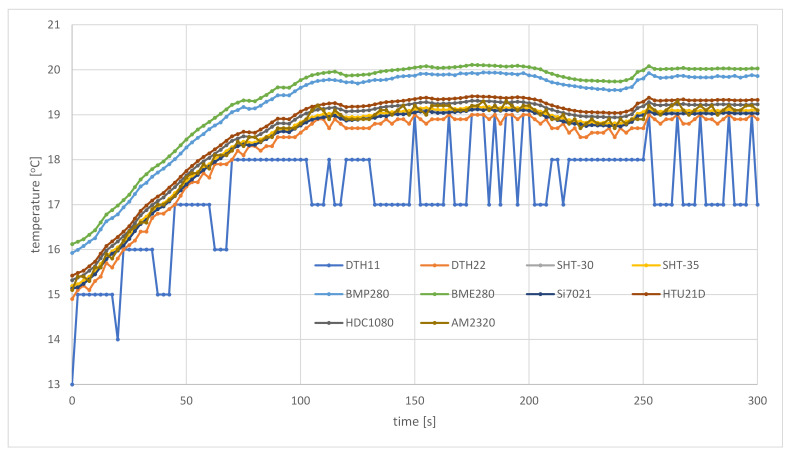
Measured temperature as a function of time of measurement, flight from a shaded place to a sunny one in the middle of May.

**Figure 15:**
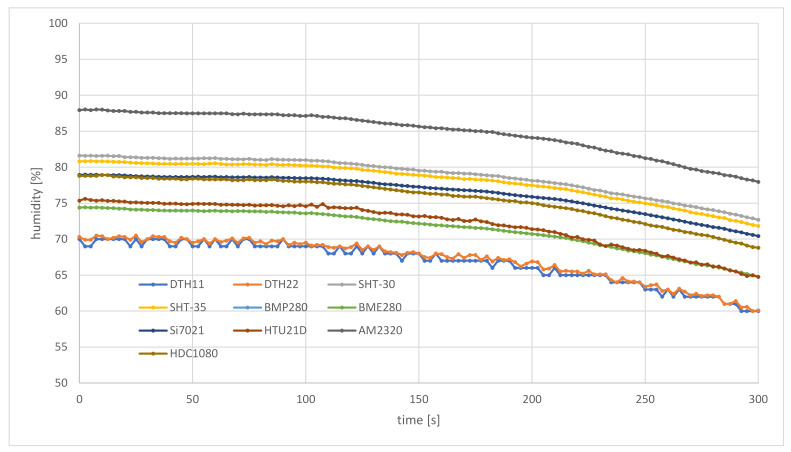
Measured humidity as a function of time of measurement, flight from a shaded place to a sunny one in the middle of May.

Figure 14 and Figure 15 compare results of temperature measurements carried out late in the morning, in the middle of May, by the air station initially located in the shade of a large building. After the first 35 m of the flight route (about 50 s of the flight), the air station flew out of the shadows into the sunlit parking lot. This caused an abrupt change of insolation, which entailed changes in other environmental parameters, including temperature and humidity. After a further 165 m of flight, the experiment was over.

The temperature in the shade of a building, measured 5 m above the ground by sensors on the air station (Figure 14), was from 15 °C (DTH11 sensor) to 16.12 °C (BME280 sensor). Although two months had passed since the vernal equinox, the ground was still not warm. As a result, this temperature was a few degrees cooler than the temperature measured 15 m above the ground (19.2 °C), at the instrument shelter located on the roof exposed to sunlight. Despite the steep change in insolation, the mixing of air masses caused the ambient temperature to increase continuously from the initial one to the temperature approximately equal to that measured by the weather station. However, the full stabilization of readings from sensors was observed only about 50 m before the end of the flight route.

In the case of the humidity (Figure 15), the mixing of air masses leads to the opposite tendency. Water-soaked soil and warm weather caused shaded places to behave as sources of air saturated with vapor, able to maintain a fixed humidity. As a result, humidity measured by the air station initially achieved 69% (the DTH11 sensor) to 87.93% (the AM2320 sensor), and stays at this level for about 90 s of flight (60 m of the flight route), when only a slight decrease in this quantity was observed. About 40 s (25 m) after the step change in insolation, Figure 15 shows a rapid decline in humidity. At the end point of the flight route, the air station flying in full sun reported humidity of 60% (DTH11) to 77.96% (AM2320). During this flight, sensors of the weather station, located in the instrument shelter on a roof, measured humidity at 69%.

**Figure 16:**
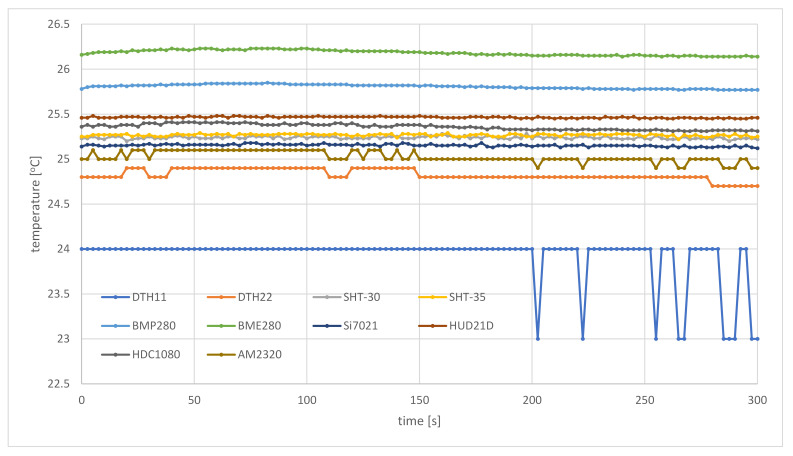
Measured temperature as a function of the time of measurement, flight in full sun in the second half of May.

**Figure 17:**
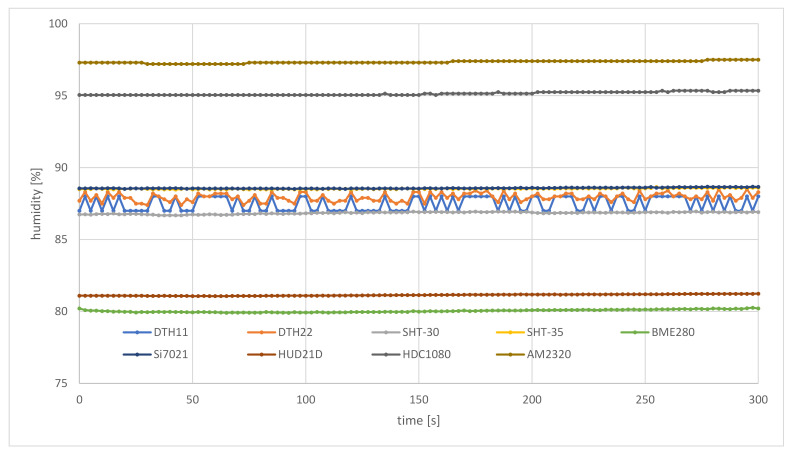
Measured humidity as a function of the time of measurement, flight in full sun in the second half of May.

Figure 16 and Figure 17 present measurements of temperature and humidity carried out by the air station following the same flight route, about a week later, a little before noon. The air station flew in stable weather conditions, with no cloud cover, in full sun. Because the IoT carrier did not go into the shadows, the sensors were exposed to direct sunlight all the time. As a result, the measurements show stable temperature and humidity, and the largest fluctuations of measurements visible in both figures result from the specificity of individual sensors. During this flight, the sensors of the SBS-WS-400 weather station reported a temperature of 24.9 °C and humidity of 82%.

From this part of the field trials, it can be concluded that the prototype weather monitoring system, based on the proposed framework, is able to measure environmental parameters at a high temporal resolution (a couple of seconds). This allowed the air station to capture local, rapid changes of environmental parameters (Figure 14 and Figure 15). In the case of a lack of change or very small changes of the measured values of temperature and humidity, data coming from the air station are also stable, which testifies to a good trade-off between measurement resolution, sensitivity and accuracy (Figure 16 and Figure 17).

### 7.3. High Spatial Resolution

**Figure 18:**
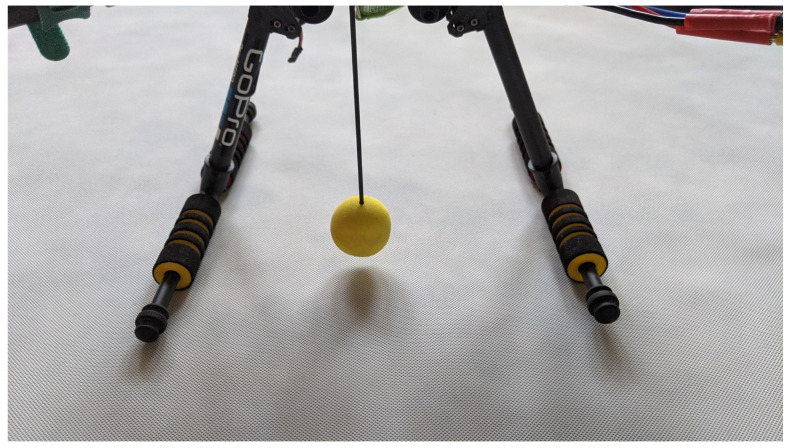
Spherical marker hung under the air station.

The proposed framework was also designed for monitoring at high spatial resolutions (of a few meters). While high temporal resolution of measurements was used during the second experiment, high spatial resolution was used in the third one, which consisted of flying the air station over a few previously established checkpoints, located at a parking lot of AGH University. Tests were carried out in the early hours of the morning, when the parking lots were empty and there were no bystanders in sight, which allowed the air station to fly at a very low altitude of 1.2 m. The air station was traveling at a speed of about 0.5 m/s (2 km/h). After arriving over a specified checkpoint the air station hovered, and the accuracy of the arrival above this point was visually checked. To facilitate visual observation, during this experiment, the air station was provided with a yellow spherical marker (Figure 18).

During the experiment, the air station flew over successive checkpoints, spaced every 5 m. Tests lasted several days, selected in terms of different Kp-indexes. This is the index of global geomagnetic activity, which affects the accuracy of positioning systems. The current value of the Kp-index is available at www.uavforecast.com (accessed on 10 June 2021).

Results show that, for low Kp-indexes, less than or equal to 2, the air station was able to fly into specified positions with an accuracy of ±0.2 m. A Kp-index of about 3 degraded the accuracy of the air station positioning over the control point to about ±0.4 m. A Kp-index close to 4 limited this accuracy to about ±0.6 m, and flying when the Kp-index is greater than or close to 5 is not recommended.

### 7.4. Visual Context

Time, in which the WebRTC application received a datum from a given sensor, the position, reported cyclicly by the positioning system and valid at the moment of receiving this datum, and the video frame captured at the moment closest to the time of reception of this datum creates, respectively, the temporal, spatial and visual context of the datum. These three contexts are captured by the WebRTC application and associated with the datum. Spatio-temporal and visual contexts are transmitted together with the datum, in an in-band (temporal context) or out-of-band (spatial and visual contexts) manner, in real-time (visual context) or near-real-time (temporal and spatial contexts). The WebRTC application running at the WMMS part of the ground station receives data and their contexts, and visualizes them on the dashboard. Received data, as well as associated temporal, spatial and video metadata, may also be stored for further processing.

**Figure 19:**
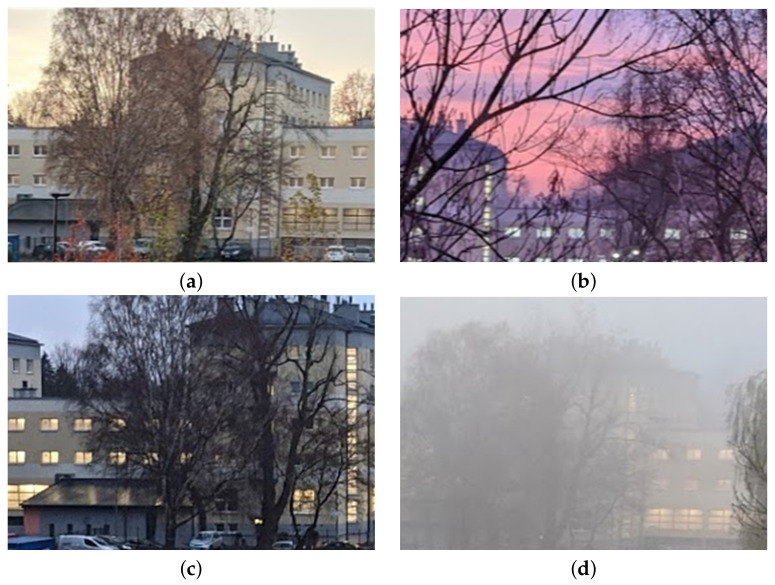
Exemplary visual contexts of transmitted data: (**a**) dawn begins; (**b**) sunrise; (**c**) cloudy day; (**d**) fog.

Figure 19 presents four exemplary visual contexts associated with sensor data during test flights of the air station. Visual information was captured using the 4K camera, listed as exemplary equipment connected to the SBC (Table 2). All pictures shown in Figure 19 were made in poor visibility conditions. As is presented in this figure, the visual part of the prototype monitoring system copes well with insufficient sunlight (both at the crack of dawn and in cloudy days) and serious problems with visibility occurred only during foggy weather.

### 7.5. AI Readiness

The proposed framework is AI-ready and the aim of the last of the described experiments was to investigate the practical possibility of running AI applications on board the air station, when the SBC is loaded with a working WebRTC application. The experiment consisted of running on the SBC both the WebRTC application and an AI application of a temperature predictor. Temperature forecasting was made on the basis of online measurements carried out by the air station. As a result, current temperature data, which were transmitted to the ground station, were, simultaneously, the input data of the AI-based predictor.

Because the proposed framework is AI-ready, it was necessary to develop modules that allow for the upload of the finished model created during the machine learning phase of the remote system. This module must be integrated with the developed solution to ensure uniform, consistent and safe communication for all elements of the system.

The AI application was written in two variants. The first one uses the well-known TensorFlow 2 library. The second one uses the TensorFlow Lite library, intended for mobile, embedded devices. The hardware platform was also tested in two variants: with and without accelerator hardware. As an accelerator hardware, the Intel Neural Compute Stick 2 was used, selected for this purpose due to its ability to co-operate with the TensorFlow library. The AI application implemented 1D Convolutional Neural Network (CNN) and Long Short-Term Memory (LSTM) to predict temperature. The LSTM is a variant of the typical Recurrent Neural Network (RNN).

During the experiment, measurements of CPU utilization and memory utilization were made. In addition, the CPU temperature was read (via specific Linux operating system variables) from the sensor factory installed in the CPU. Statistics of these performance metrics as well as total execution time (averaged over 5 executions) are included in Table 6. This table presents the comparison of performance metrics of the SBC running the AI application written using the classic TensorFlow library, the SBC running the application written using the TensorFlow Lite library, and the SBC co-operated with the Intel Neural Compute Stick 2 accelerator when the application written using the TensorFlow Lite library was run.

As is presented in Table 6, the simultaneous work of the WebRTC application working in UAV mode and the AI-based temperature predictor caused the average CPU utilization to be 85% in the case of the TensorFlow library, 96% in the case of the TensorFlow Lite library, and 52% if the application based on the TensorFlow Lite library was co-operating with the accelerator hardware. Maximum CPU utilization was 95%, 99%, and 70%, respectively. As a reminder, the average CPU utilization of the Raspberry Pi 4 Model B, loaded only with the WebRTC application, was 32%, and the maximum was 70% (Table 2).

It is worth noting that less CPU utilization by the application based on the TensorFlow library, when compared to the same application using the TensorFlow Lite library, is caused by the less efficient code of the TensorFlow that takes longer to execute (more than two times longer than the TensorFlow Lite code). The TensorFlow Lite is optimized for the Advanced Reduced Instruction Set Computing Machine (Advanced RISC Machine, ARM) architecture of microprocessors. As a result, it executes the maximum number of Raspberry Pi instructions per clock cycle, which leads to better CPU utilization and smaller execution times.

Generally, accelerator hardware able to co-operate with the used programming library is necessary for the hassle-free running of AI applications on the air station. Without hardware acceleration, the temperature of the Raspberry Pi grew rapidly and, when it exceeded 76 °C, a thermal throttling of this CPU was observed—the CPU reduced the clock frequency to prevent overheating. This led to the reduction of resources available to both applications (AI and WebRTC) and, as a result, to a significant reduction in their performance.

## 8. Use Cases

The proposed UAV-Based and WebRTC-Based open universal framework was intended for fast prototyping of flying IoT systems that may be used to monitor urban and industrial areas. Typical usage of such systems include: monitoring of parking lots (both in terms of security and air quality), traffic monitoring, security monitoring, weather monitoring, fire monitoring, monitoring of power lines and pipelines. Realization of such tasks requires that the framework must be equipped with a set of sensors, customized for the specific tasks of monitoring, accompanying with a UAV camera for surveillance purposes. In the times of the COVID-19 pandemic, systems based on the proposed framework may realize such tasks as measuring the temperatures of people in the open or detecting persons that break face covering orders.

The proposed framework can be used for building IoT systems that autonomously carry out a number of operations with the use of the AI hardware accelerator being a part of this framework. As an example, it can recognize license plates of cars in a parking lot, and retrieved data can be used as metadata for indexing data coming from sensors and video information coming from the UAV’s camera. It is also possible to detect anomalies in road and pedestrian traffic. Such anomalies may result, for example, from road accidents or various random events, or security threats. Other kinds of anomalies can be used for image-based plant disease detection, carried out on city squares and parks. On-board AI that performs edge computing may be also used for weather nowcasting, intended to predict (with a tolerable degree of accuracy) weather in very short time horizons.

Because the maximum load of the used UAV is up to 1 kg, the framework must be able to carry the equipment needed for these tasks, even if the measured weight of the sensors will be larger than read from specifications. For example, the HTU21D weights about 2 g (according to specification: 1.8 g) and BME280 also weighs about 2 g (according to specification: 1.7 g). Sensors are mounted on boards and their weights (both measured and specified value) are given together with their boards.

In addition, the measured power consumption was small enough to assure work of the prototype based on the proposed framework. For example, the HTU21D sensor during typical measurements of temperature and humidity does not exceed 420 μA, and the energy consumption of the BME280 performing temperature measurements does not exceed 210 uA (without measurements of atmospheric pressure) and 340 μA (with measurements of pressure). The measured energy consumption of the Intel NCS2 was from 110 mA to 420 mA. In addition, the measured energy consumption of the Raspberry Pi 4 B was from 580 mA to 890 mA. It is worth noting that all measurements were carried out in typical working conditions for the framework, i.e., for the frequencies of measurements that were maximum or close to maximum. In such conditions, the energy consumption of the GNSS SIM700E module working as the GNSS was about 31 mA. The Manta MM9359FS camera had an energy consumption of about 120 mA.

## 9. Conclusions

Nowadays, UAV-based monitoring systems face the challenges of smart cities and industrial areas, as well as the requirement for high temporal and spatial resolution of measurements. On the other hand, there is tendency to load the IoT hub with additional computing, which embodies the idea of edge computing, as well as AI-supported computing. The proposed open, universal framework was designed for fast prototyping or the building of a short series of specialized flying monitoring systems that meet these challenges. The air station combines a custom assembled UAV carrying an IoT and having WebRTC communication. The latter is important because it is common technology for both real-time video and reliable data transmission, which leads to (thanks to extensive congestion control) near-real-time transmission of data coming from sensors and contextual communication. The proposed framework is AI-ready. It includes AI accelerator hardware that allows an IoT communication hub placed on the UAV to perform tasks of AI-supported computing.

The presented open universal framework for rapid prototyping of UAV-based monitoring systems was evaluated using the example of a weather monitoring system, assembled with the use of the proposed framework and 10 low-cost weather sensors that measure temperature and relative humidity. Tests and fields trials were carried out at the campus of the AGH University of Science and Technology, Poland. Their results show that the WebRTC application running on the Raspberry Pi 4 Model B processor is fast enough to serve fast-response sensors, and that the prototype was able to measure environmental parameters at both high temporal resolution (a couple of seconds) and high spatial resolution (a few meters). Moreover, analysis of the visual part of this system shows that it is able to perform its tasks even in poor visibility conditions (insufficient sunlight). Analysis of AI-readiness shows that the use of AI accelerator hardware makes this framework AI-ready.

Future avenues for research include going towards multiple air stations served by a single ground station, and, conversely, a single air station that flies through areas controlled by different ground stations and connects to each of them. This would allow the air station to accept requests from each of the ground stations and to perform tasks specific to a given area.

## Figures and Tables

**Table 1:** Comparison of UAV-based IoT frameworks.

Authors	Type of UAV	Application	Main Features	Comments
Alfattani et al. (2019) [56]	UAV swarm (unknown)	collection data from multiple IoT	cooperation UAVs with wireless sensor networks (WSNs)	optimization of location of multiple UAVs
Gu et al. (2018) [55]	UAV (hexacopter)	air monitoring system	modular design techniques	multiple programing
Horstrand et al. (2019) [57]	UAV (hexacopter)	agriculture monitoring	advance processing images	commercial drone with designed vision subsystem
Kim et al. (2018) [58]	UAV swarm (heterogeneous UAVs)	monitoring smart city and extensive ocean	cooperation of drones	two models of cooperating drones one applied for a city, and the second for ocean
Lagkas et al. (2018) [4]	UAV (unknown)	general purpose IoT	security issues	usage of multiple protection solution
Liu et al. (2020) [59]	UAV swarm (unknown)	air quality monitoring	federated learning	Graph Convolutional neural network-based Long Short-Term Memory (GC-LSTM) model to provide real-time federated learning
Matese and Di Gennaro (2018) [60]	UAV (hexacopter)	agriculture monitoring	multiple sensors	vision analysis of multiple types of plants
Motlagh et al. (2016) [23]	UAV (unknown)	general purpose IoT	multicriterial optimization of UAV	use Linear Integer Problem (LIP) optimization solution
Pircher et al. (2017) [61]	UAV (fixed-wing and rotary-wing)	precision agriculture	integration of sensors	direct to ground station digital (telemetry 433 MHz) and analog transmission
Sandino et al. (2020) [62]	UAV (quadcopter)	search and rescue missions	low cost system	GNSS-denied environments
Sokač et al. (2016) [63]	UAV (quadcopter)	air monitoring	low cost system	sensor data transmission using telemetry 433 MHz

**Table 2:** Comparison of performance metrics of three types of Raspberry Pi.

	Raspberry Pi		
	3 Model B	3 Model B+	4 Model B
average usage of memory	86% (of 1 GB)	84% (of 1 GB)	51% (of 2 GB)
average CPU utilization	96%	76%	32%
maximum observed CPU utilization	100%	89%	70%

**Table 3:** Devices used in the exemplary prototype.

Device	Type	Comments
4K camera	Manta MM9359FS	live streaming via USB or recording to a local storage device
weather sensors	DTH11	temperature and humidity
	DTH22	temperature and humidity
	SHT-30	temperature and humidity
	SHT-35	temperature and humidity
	BMP280	temperature and atmospheric pressure
	BME280	temperature, humidity, and atmospheric pressure
	Si7021	temperature and humidity
	HTU21D	temperature and humidity
	HDC1080	temperature and humidity
	AM2320	temperature and humidity
positioning module	Waveshare SIM7000E	NB-IoT LTE GPS HAT
AI accelerator hardware	Intel NCS2	dedicated neural compute engine and Vision Processing Unit (VPU)

**Table 4:** Comparison of sensor response time (temperature).

Sensor Type	Time			
	Min	Max	Mean	Nominal
DTH11	1.1 s	1.2 s	1.14 s	1.0 s
DTH22	2.1 s	2.4 s	2.2 s	2.0 s
sHT-30	4.5 ms	6.5 ms	5 ms	4.5 ms
sHT-35	4.8 ms	6.4 ms	5.1 ms	4.5 ms
BMP280	9.7 ms	14 ms	12 ms	9.7 ms
BME280	11.5 ms	14 ms	12 ms	11.5 ms
si7021	2.4 ms	4.2 ms	3.5 ms	2.4 ms
HTU21D	11 ms	14 ms	12 ms	11 ms
HDC1080	3.65 ms	4.1 ms	4.0 ms	3.65 ms
AM2320	2.1 s	2.3 s	2.2 s	2.0 s

**Table 5:** Comparison of sensor response time (humidity).

Sensor Type	Time			
	Min	Max	Mean	Nominal
DTH11	1.1 s	1.2 s	1.14 s	1.0 s
DTH22	2.1 s	2.4 s	2.2 s	2.0 s
sHT-30	4.6 ms	6.5 ms	5.1 ms	4.5 ms
sHT-35	4.8 ms	6.5 ms	5.2 ms	4.5 ms
BME280	12.5 ms	16 ms	13 ms	12.5 ms
si7021	3.7 ms	5 ms	4.1 ms	3.7 ms
HTU21D	4 ms	6 ms	5 ms	4 ms
HDC1080	3.85 ms	4.2 ms	4.1 ms	3.85 ms
AM2320	2.1 s	2.4 s	2.2 s	2.0 s

**Table 6:** Comparison of performance metrics.

	Raspberry Pi TF	Raspberry PI TF Lite	Raspberry PI and Intel NCS2 TF Lite
average usage of memory	72%	64%	65%
average CPU utilization	85%	96%	52%
maximum observed CPU utilization	95%	99%	70%
average CPU temperature	57 °C	61 °C	51 °C
maximum CPU temperature	73 °C	77 °C	62 °C
execution time	223 ms	110 ms	41 ms

## Data Availability

Not applicable.

## Abbreviations

The following abbreviations are used in this manuscript:

3S	three-cell
4S	four-cell
5S	five-cell
ADC	Analog to Digital Converter
AI	Artificial Intelligence
API	Application Programming Interface
BEC	Battery Eliminator Circuit
BLE	Bluetooth Low Energy
CCC	Command and Control Console
CPU	Central Processing Unit
DIY	Do It Yourself
GCC	Google Congestion Control
GNSS	Global Navigation Satellite System
GPIO	General Purpose Input–Output
GPU	Graphics Processing Unit
GUI	Graphical User Interface
HDMI	High Definition Multimedia Interface
HTML5	Hypertext Markup Language version 5
IO	InputOutput
I2C	Inter-Integrated Circuit
IEEE	Institute of Electrical and Electronics Engineers
FHSS	Frequency-Hopping Spread Spectrum
FPV	First Person View
IoT	Internet of Things
LiPo	Lithium Polymer
LTE	Long Term Evolution
MCU	Microcontroller Unit
ML	Machine Learning
MQTT	Message Queue Telemetry Transport
NCS2	Neural Compute Stick 2
NSS	Navigation Satellite System
OSI	Open Systems Interconnection
OSM	OpenStreetMap
OSMC	Open Source Media Center
QoE	Quality of Experience
QoS	Quality of Service
SBC	Single-Board Computer
SDRAM	Synchronous Dynamic Random Access Memory
PAL	Phase Alternating Line
RTP	Real-time Transport Protocol
SCTP	Stream Control Transmission Protocol
SDIO	Secure Digital Input Output
SPA	Single Page Application
SPI	Serial Peripheral Interface
SRAM	Static Random Access Memory
TFRC	TCP-Friendly Rate Control
UAV	Unmanned Aerial Vehicles
UART	Universal Asynchronous Receiver/Transmitter
URL	Uniform Resource Locator
USB	Universal Serial Bus
VPU	Vision Processing Unit
WebRTC	Web Real-Time Communications
WiFi	Wireless Fidelity
WMMS	WebRTC Multimedia and Monitoring Station
